# A challenging case of solitary fibrous tumor of the orbit in an anemic patient

**DOI:** 10.22336/rjo.2024.82

**Published:** 2024

**Authors:** Sonali Vinay Kumar, Manoj Gopal Madakshira, Vinay Kumar, Alok Sati, Natasha Vinay Kumar, Sandeepan Bandopadhyay

**Affiliations:** 1 Command Hospital Eastern Command, Kolkata, India,; 2 JIS School of Medical Science and Research, Howrah, India,; 3471 Field Hospital, Arunachal Pradesh, India,; 4 Department of Medicine, Sri Devaraj Urs Medical College, Kolar, India,

**Keywords:** tumor, challenging, histopathological, immunohistochemical, SFT = Solitary fibrous tumor, MRI = magnetic resonance imaging, CT = computed tomography

## Abstract

We report a problematic case of solitary fibrous tumor of the orbit in an anemic patient who presented with painless, progressive outward protrusion of the right eye. Magnetic resonance imaging (MRI) orbit and brain with contrast showed a well-defined intraconal lesion in the superomedial aspect of the right orbit. The lesion was excised using a vertical eyelid split orbitotomy with minimal blood loss. Histopathological and immunohistochemical examination showed the features of a solitary fibrous tumor. This case highlighted that with careful surgical planning, total removal of a large vascular tumor could be done with less bleeding, especially in an anemic patient in whom an iota of hemorrhage was life-threatening.

## Introduction

A solitary fibrous tumor (SFT) of the orbit is sporadic, with approximately 154 cases reported in the literature [1-3]. SFT is a spindle-cell tumor of mesenchymal origin, initially described in the pleura by Klemperer and Rabin in 1931 [4]. Extrapleural SFT has been recognized recently in the pericardium, peritoneum, lung, liver, nasal cavities, mediastinum, thyroid, parotid gland, and orbit [5]. The orbital solitary fibrous tumor was first described by Dorfman et al. and Westra et al. in 1994 [6,7]. Solitary fibrous tumors are often misdiagnosed as they share similar clinical and microscopic features with other mesenchymal tumor/fibroblastic tumors such as hemangiopericytoma, angiofibroma, nerve sheath tumor, meningioma, fibrous histiocytoma, and cavernous hemangioma. Hence, immunohistochemical features play a vital role in the definitive diagnosis of the entity. Being vascular, the treatment of fibroblastic tumors is complex as torrential bleeding can occur during the removal of this tumor, which can cause substantial blood loss in the patient. In an anemic patient, any bleeding can be detrimental to the health of the patient and life-threatening; hence, the orbital tumor should be meticulously removed. Herein, we report an arduous case of solitary fibrous tumor of the orbit in a nutritional anemic patient, which was managed successfully as complete excision of the tumor could be achieved without causing significant extravasation of blood.

### Case details

**Fig. 1:**
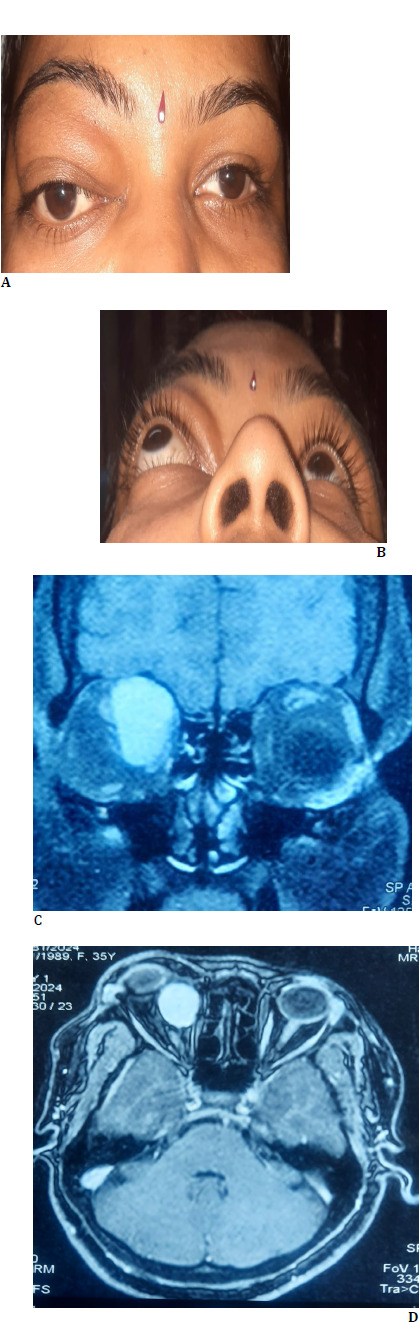
**A, B**: Clinical photograph demonstrating right eye proptosis in the primary and worm’s view positions. **C, D**: MRI orbit and brain image showing a well-defined intraconal lesion along the superomedial aspect of the right orbit in the coronal and axial scan

A 36-year-old female patient presented with painless protrusion of the right eye and downward displacement of the eye for 5 years. The patient did not give a history of visual disturbance/pain/double vision. The patient also did not provide h/o trauma or prior ocular surgery. The patient’s personal and family history were unremarkable. Clinical examination showed proptosis of the right eye as Hertel exophthalmometer measurement at 110 base revealed 26 mm in the right and 18 mm in the left eye. It also showed the right eye hypoglobus (**Fig. 1 A, B**). Visual acuity in both eyes was 20/20. Ocular movements were full and free in both eyes. Pupils were round, central, and reacting to light. Intraocular pressure with the noncontact tonometer was 18 mm in the right eye and 16 mm in the left eye. Anterior and posterior segment evaluations in both eyes were unremarkable. Magnetic resonance imaging (MRI) orbit showed a well-defined ovoid/round lesion in the intraconal compartment of the right orbit along its superomedial aspect about 25x17x25 mm in size (**Fig. 1 C, D**). The lesion was abutting and displacing the right globe inferolateral to the right side. The provisional diagnosis of RE soft tissue tumor with vascular or connective tissue origin was made based on clinical and MRI image features. The general and systemic examinations were insignificant. There was no regional lymphadenopathy. Hematological investigation showed a Hb level of 8 gm/dl. A peripheral blood smear study showed a microcytic, normocytic hypochromic RBC series. Bone marrow aspiration cytology showed mild hypercellular marrow, adequate trilineage hematopoiesis, and mild erythroid hyperplasia.

**Fig. 2:**
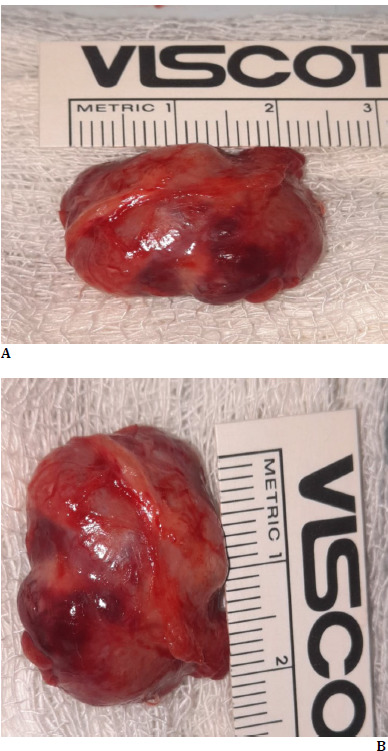
**A, B**: Clinical photograph showing the specimen of excised mass (about 25x24 mm in size)

The patient was subjected to anterior orbitotomy, and the tumor was removed via a vertical lid split orbitotomy. Intraoperatively, the tumor was found to be firm and vascular. It was pinkish and not lobulated. It showed extensive adhesion to the surrounding muscular structure. Careful dissection was done to avoid damage to critical structures in the orbit. After all fibrous adhesions between the tumor were released, the cancer was released in toto. Haemostasis was achieved with radiofrequency cautery. The lid incision was repaired in a way we repair full-thickness lid margin laceration. One unit of blood (500 ml) was administered during surgery. The lesion was about 25x24 mm and was sent for histopathological examination (**Fig. 2 A, B**). Post-operatively, the patient was found stable and started on oral antibiotics and anti-inflammatory medications.

**Fig. 3:**
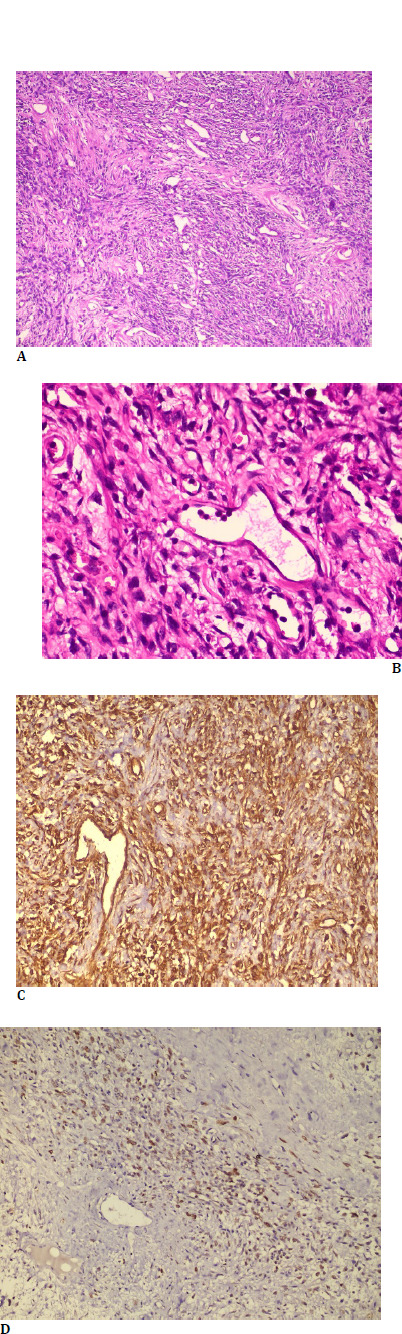
**A**: Hematoxylin and Eosin stain (100x magnification) showing monomorphic spindle cell tumor arranged in bundles and a pericytoma pattern around the vascular spaces (star) **B**: H & E stain (400x magnification) showing spindle cells around an endothelial cell lined vascular space showing characteristic staghorn/branched appearance (star). **C**: Immunohistochemistry (200x magnification) with Vimentin showing the cytoplasmic staining pattern in the spindle cells and the endothelial cells lining the vascular space (star). **D**: Immunohistochemistry (200x magnification) with STAT 6 showing the nuclear staining pattern in the spindle cells with sparing of the endothelial cells lining the vascular space (star)

Histopathological examination showed a well-circumscribed lesion composed of predominantly cellular and a few hypocellular areas. The lesion comprised spindle cells arranged in vague fascicles with interspersed varying-sized hyalinized blood vessels (**Fig. 3 A, B**). No evidence of mitosis, nuclear pleomorphism, or necrosis was observed.

Immunohistochemistry showed the tumor cells to be positive for Vimentin (cytoplasmic), CD34, SMA, and STAT6 while being negative for S100p (**Fig. 3 C, D**). Based on histopathology and immunohistochemistry features, a final diagnosis of solitary fibrous tumor was made.

**Fig. 4:**
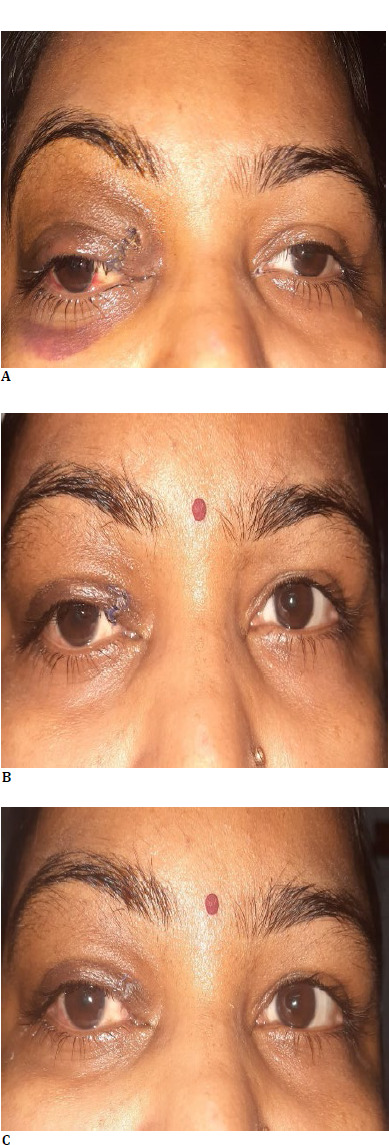
**A-C**: Postoperative image showing resolution in proptosis at 1-week, one month, and 6 months, with no sign of recurrence

The patient was followed up for 6 months. During this period, proptosis resolved entirely, and no sign of recurrence was found (**Fig. 4 A-C**).

## Discussion

Accurately diagnosing a well-defined orbital mass based on clinical and MRI images is challenging as most fibroblastic, vascular, and nerve sheath tumors present with similar features. Mesenchymal tumors account for 5 to 8% of orbital neoplasms. Haemangiomas and fibrous histiocytomas are the most common primary orbital mesenchymal tumors. Histologically, SFT was thought to be of mesothelial origin but is now considered to have a mesenchymal and possibly fibroblastic origin. The awareness about orbital SFT has increased, and more cases are being reported since its first description in orbit in 1994 by Westra et al. Advanced immunohistochemical techniques have helped in this regard. Immunohistochemical studies, mainly positive for CD 34, Vimentin, and bcl-2, played an essential role in definitively diagnosing a solitary fibrous tumor.

Orbital SFT can involve any orbital part, including the lacrimal gland fossa, lacrimal sac, conjunctiva, and sclera. It is usually seen in people aged 20 to 76, mainly middle-aged adults with no gender affiliation. The typical presentation in this entity is unilateral, slowly progressive, painless proptosis with well-defined, strongly enhancing lesions on Computed Tomography (CT) and MRI [1,2,3,8]. It is considered a benign lesion, but a local invasion and recurrence can occur following an incomplete initial excision. The definitive treatment of an SFT of the orbit is a complete surgical excision with long-term follow-up.

Orbital solitary fibrous tumors in pregnant women have been reported in the literature, but the occurrence of this entity with other systemic conditions has not been mentioned in any studies [9,10]. The manifestation of SFT of the orbit in anemic patients was found in the current study, which is reported for the first time through this case report. The dilemma was whether the risk of operating on the vascular tumors in anemic patients was worth it or not. The main worry was intraoperative bleeding, which can further aggravate anemia in this patient and put her life at risk. However, with meticulous planning and by adopting the appropriate surgical technique, a prominent vascular tumor was entirely removed without causing significant blood loss and endangering the life of the patient in our case.

This case underscores the need for a multidisciplinary approach to effectively tackling a vascular orbital tumor in anemic patients.

## Conclusion

Though a solitary fibrous tumor of the orbit is a rare entity, it must be considered in the differential diagnosis of a well-defined orbital mass. Histopathological and immunohistochemical examination plays a crucial role in appropriately diagnosing this lesion. With diligent surgical planning, complete excision of vascular tumors is possible with less bleeding in patients affected by systemic comorbidities.
